# Analysis of the Putative Remains of a European Patron Saint–St. Birgitta

**DOI:** 10.1371/journal.pone.0008986

**Published:** 2010-02-16

**Authors:** Martina Nilsson, Göran Possnert, Hanna Edlund, Bruce Budowle, Anna Kjellström, Marie Allen

**Affiliations:** 1 Rudbeck Laboratory, Department of Genetics and Pathology, Uppsala University, Uppsala, Sweden; 2 Forensic Unit, Regional Criminal Investigation Department, Stockholm County Police, Stockholm, Sweden; 3 The Ångström Laboratory, Department of Engineering Sciences, Uppsala University, Uppsala, Sweden; 4 FBI Laboratory, Quantico, Virginia, United States of America; 5 Department of Forensic and Investigative Genetics, University of North Texas Health Science Centre, Ft Worth, Texas, United States of America; 6 The Wallenberg Laboratory, Department of Archaeology and Classical Studies, Stockholm University, Stockholm, Sweden; Freie Universitaet Berlin, Germany

## Abstract

Saint Birgitta (Saint Bridget of Sweden) lived between 1303 and 1373 and was designated one of Europe's six patron saints by the Pope in 1999. According to legend, the skulls of St. Birgitta and her daughter Katarina are maintained in a relic shrine in Vadstena abbey, mid Sweden. The origin of the two skulls was assessed first by analysis of mitochondrial DNA (mtDNA) to confirm a maternal relationship. The results of this analysis displayed several differences between the two individuals, thus supporting an interpretation of the two skulls not being individuals that are maternally related. Because the efficiency of PCR amplification and quantity of DNA suggested a different amount of degradation and possibly a very different age for each of the skulls, an orthogonal procedure, radiocarbon dating, was performed. The radiocarbon dating results suggest an age difference of at least 200 years and neither of the dating results coincides with the period St. Birgitta or her daughter Katarina lived. The relic, thought to originate from St. Birgitta, has an age corresponding to the 13^th^ century (1215–1270 cal AD, 2σ confidence), which is older than expected. Thus, the two different analyses are consistent in questioning the authenticity of either of the human skulls maintained in the Vadstena relic shrine being that of St. Birgitta. Of course there are limitations when interpreting the data of any ancient biological materials and these must be considered for a final decision on the authenticity of the remains.

## Introduction

During the High Middle Ages, a remarkable woman, Birgitta Birgersdotter (1303–1373) became known for her many revelations, prophecies and pilgrimages. She was canonized as a Roman Catholic saint in 1391. In 1999, Pope Johannes Paulus II in Rome assigned St Birgitta as a patron saint for Europe. St. Birgitta died 1373 in Rome and her final wish was that her remains should be brought back to Sweden. Following her death the remains were boiled to remove the soft tissue. The bones were transported from Rome to Vadstena and placed in a relic casket in 1374. At times, pieces of the St. Birgitta relics were given to churches and monasteries, but also to prominent persons like the Pope, the German-Roman Emperor and the King of England [1]. Today the shrine contains two skulls and 23 other human bones, relics from different individuals. The skulls are the putative remains of St. Birgitta and her daughter, the beatified Katarina (1331–1381). The remains of Katarina were first buried and thereafter recovered and placed in the shrine in 1489. In addition to the two skulls in the shrine in Vadstena today, a third skull has been mentioned as present until 1645 when it was stolen. The third skull has previously been excluded as being from St. Birgitta or Katarina based on morphological characteristics (mainly age) [1].

**Figure 1:**
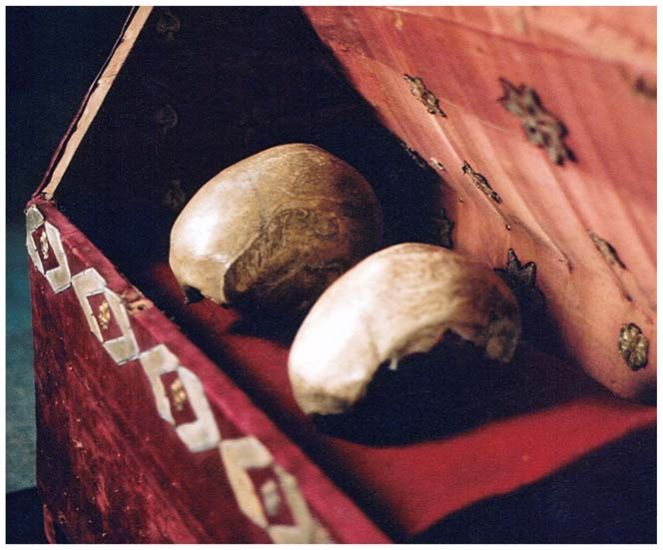
Picture of the relics. The putative skull of St. Birgitta (skull A) to the right and the putative skull of Katarina (skull B) to the left. (Photograph: Hans Lundberg).

Following a thorough anthropological and archaeological investigation, performed in 1952 by Bygdén, Gejvall and Hjortsjö, the content of the relic shrine was described in detail. Their assumptions and conclusions were based on criteria such as sex, the presumed age of death, and the colour and consistency of the bones. They concluded that both skulls most likely are from females. One of the two skulls is from a person deceased at age 60–70, consistent with the age at death of St. Birgitta (Figure 1, Skull A). The other skull is from someone who deceased at a younger age of approximately 50–55 years, consistent with that of Katarina (Figure 1, Skull B) [1]. In present time, the parish of Vadstena requested a DNA-based analysis to provide additional information regarding the authenticity of the two skulls. Since the skulls are reputed to be from mother and daughter, analysis of the maternally inherited mitochondrial DNA (mtDNA) from the skulls and a maternal relative as a reference would be ideal. However, a maternal relative could not be found. Therefore, the maternal lineage relationship hypothesis was tested by comparing hypervariable regions of mtDNA between the two skulls.

## Results

The putative skulls of St. Birgitta and Katarina were compact and appeared to be very well preserved for their presumed age. An anthropological investigation of the sexually dimorphic cranial traits was performed. The sex characteristic features of skull A and B were gracile indicating that they are from women. An oval concave depression was observed in the anthropological analysis of skull A. This characteristic was also noticed in previous investigations of the skull, and can be caused by a benign tumour (meningioma) [1], [2]. Although the discovery of soft-tissue cancer is rare in archaeological samples, a case of meningioma has been diagnosed in a 365 000 years old skull from Steinheim/Murr, Germany [3].

**Figure 2:**
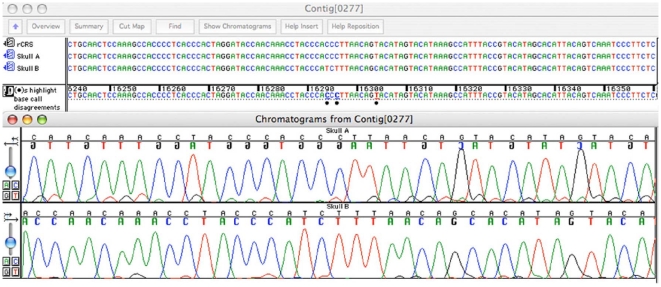
Sanger sequence chromatograms from skull A and B, illustrating three of the sequence differences (16294, 16296 and 16304) between the skulls. The sequences were compared with rCRS [33].

The DNA extractions from the skulls resulted in relatively high yields of DNA and the following analyses of mtDNA revealed sequence data of high quality. The DNA extractions were performed by two different analysts, at separate occasions, from each skull using two different protocols. Standard precautions to prevent contamination were taken, and in addition, one of the protocols involved the use of sodium hypochlorite (NaOCl) to destroy exogenous contaminants before demineralisation of the bone [4], [5]. The mtDNA hypervariable regions (HVI and HVII) were amplified and sequenced from the two extracts from each individual. In total, 60 PCR products (out of 96 reaction set-ups) were successfully amplified and sequenced. The sequence analysis of the hypervariable regions revealed sequence differences at six nucleotide positions (excluding the HVII c-stretch variation) between the two skulls, of which five were found in HVI and one in HVII (Table 1 and Figure 2). This number of differences is similar to the average number of nucleotide differences between two unrelated European Caucasians [6]. According to guidelines applied in forensic mtDNA sequencing, the presence of two or more nucleotide differences between two samples is sufficient to exclude a common source or that two individuals originate from the same maternal lineage [7], [8]. Consequently, the mtDNA results reject the hypothesis of a mother-daughter relationship between the two individuals in the shrine. In addition, a DNA based sex determination was performed using Pyrosequencing of the Amelogenin gene. Both skull samples demonstrated a 6 bp deletion in the Amelogenin gene, confirming female individuals. Thus, the DNA data is in concordance with the anthropological sex determination performed previously.

**Table 1:** Results of mtDNA Sanger sequencing, Pyrosequencing and age determination.

	HVI						HVII					Coding				
	16126	16189	16294	16296	16304	16362	73	239	263	309.1	315.1	3010^2^	16519^2^	^δ13^C (‰ VPDB)	^14^C age (BP)	^14^C dating (cal AD)^4^
**rCRS** 1	T	T	C	C	T	T	A	T	A	:	:	G	T			
**Skull A** 1	T	C	C	C	T	T	A	T	G	:	C	A	C	−18.5^3^	791±17^3^	1220–1265 (1σ) 1215–1270 (2σ)
**Skull B** 1	C	T	T	T	C	T	G	T	G	C	C	G	C	−20.3	295±45	1510–1660 (1σ) 1470–1670 (2σ)
**Analyst 1** 1	T	T	C	C	T	T	A	T	G	C	C	G	C			
**Analyst 2** 1	T	T	C	C	T	C	A	C	G	C	C	G	C			

1 rCRS, revised Cambridge Reference Sequence [33]. Skull A, sequence from the putative skull of St. Birgitta 1303–1373. Skull B, sequence from the putative skull of Katarina 1331–1381. Analyst 1, sequence from Analyst 1. Analyst 2, sequence from Analyst 2.

2 Pyrosequencing results of mtDNA coding regions.

3 Mean value of three different sample preparations from two different bone samples from the skull A.

4 σ refers to the standard deviation, where 1σ corresponds to 68.2% probability and 2σ to 95.4% probability according to the OxCal v3.10.

**Figure 3:**
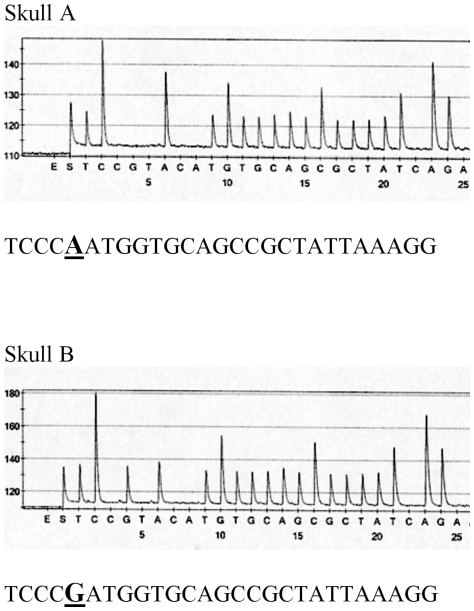
Pyrosequencing results at position 3010 in the mtDNA coding region. An A/G difference is displayed between the two skulls. The upper sequence from skull A is TCCC**A**ATGGTGCAGCCGCTATTAAAGG and the lower sequence from skull B is TCCC**G**ATGGTGCAGCCGCTATTAAAGG.

To exclude that the results could be due to contamination by any of the two analysts performing the analysis, their DNA was sequenced. One of the two analysts had an mtDNA type differing only at position 16189 (excluding c-stretch variation) from skull A (Table 1), and a single mtDNA difference (excluding the c-stretch in HVII) is not sufficient for exclusion in forensic analysis [7], [8]. This finding is not surprising since the sequence of analyst 1 is the most common type among Caucasians (7%) [9]. However, to dispel the possibility that the result may be due to contamination from the analyst, sequencing of two regions in coding mtDNA, containing the variable positions 3010G/A and 16519T/C, was performed [10]. The analysis is based on pyrosequencing and resulted in one additional difference in the coding region 3010G/A between the analyst and skull A (Table 1). Furthermore, skull A and B displayed an additional difference in the 3010 position (Table 1 and Figure 3).

An interesting observation revealed by DNA analysis was that the samples from the two skulls yielded different results with respect to the number of positive PCR reactions as well as yield of product estimated by agarose electrophoresis. The mtDNA hypervariable regions (HVI and HVII) were amplified in fragments of two different lengths. The HVI fragments were 221 and 440 bp long, and the HVII fragments were 243 and 415 bp. A total of 48 different amplification attempts were performed for each of the individuals (24 shorter and 24 longer PCR reactions). In total, 24 and 36 PCR products were positive from skull A and B extracts, respectively. The longer HVI and HVII fragments yielded 3 positive PCR reactions for skull A and 13 for skull B. For the shorter fragments, 21 positive PCR reactions were obtained for skull A and 23 for skull B (Table 2). A notable difference in success rate was thus seen between the two skulls for the longer fragments (13% for skull A and 54% for skull B). The samples show an inverse relation between success rate and fragment length, indicating a certain degree of degradation.

**Table 2:** Sanger sequencing results of 60 PCR products obtained amplifying mtDNA HVI and HVII.

	HVI		16049	16126	16189	16294	16295	16296	16304	Size^2^		HVII		73	239	263	309.1	315.1	368	Size^2^
Sample	Coverage	rCRS^1^	G	T	T	C	C	C	T		Sample	Coverage	rCRS^1^	A	T	A	:	:	A	
**Skull A** 1	16012–16382	PCR 1	G	T	C	C	C	C	T	L	**Skull A** 1	35–397	PCR 1	A	T	G	:	C	A	L
	16158–16292	PCR 2			(T/C)					S		53–407	PCR 2	A	T	G	:	C	A	L
	16161–16192	PCR 3			C					S		67–263	PCR 3	A	T	G				S
	16180–16275	PCR 4			C					S		67–244	PCR 4	A	C/T					S
	16180–16320	PCR 5			C	C	C	C	T	S		67–263	PCR 5	A	T	G				S
	16180–16320	PCR 6			C	C	C	C	T	S		67–244	PCR 6	A	T					S
	16180–16320	PCR 7			C	C	C	C	T	S		67–244	PCR 7	A	T					S
	16180–16322	PCR 8			C	C	(T/C)	C	T	S		68–263	PCR 8	A	T	G				S
	16184–16287	PCR 9			C					S		69–263	PCR 9	A	T	G				S
	16184–16289	PCR 10			(T/C)					S		69–263	PCR 10	A	T	G				S
	16184–16289	PCR 11			C					S		101–263	PCR 11		T	G				S
	16184–16289	PCR 12			C					S	**Skull B** 1	35–399	PCR 1	G	T	G	C	C	A	L
	16184–16291	PCR 13			C					S		35–371	PCR 2	G	T	G	C	C	A	L
**Skull B** 1	15991–16342	PCR 1	G	C	T	T	C	T	C	L		35–399	PCR 3	G	T	G	C	C	A	L
	15991–16382	PCR 2	G/A	C	T	T	C	T	C	L		35–397	PCR 4	G	T	G	C	C	A	L
	16014–16382	PCR 3	G	C	T	T	C	T	C	L		35–397	PCR 5	G	T	G	C	C	A	L
	16020–16382	PCR 4	G	C	T	T	C	T	C	L		53–407	PCR 6	G	T	G	C	C	A/G	L
	16082–16390	PCR 5		C	T	T	C	T	C	L		53–407	PCR 7	G	T	G	C	C	A	L
	16082–16390	PCR 6		C	T	T	C	T	C	L		67–263	PCR 8	G/A	T	G				S
	16153–16320	PCR 7			T	T	C	T	C	S		67–263	PCR 9	G	T	G				S
	16153–16320	PCR 8			T	T	C	T	C	S		67–263	PCR 10	G	T	G				S
	16153–16320	PCR 9			T	T	C	T	C	S		67–263	PCR 11	G	T	G				S
	16153–16320	PCR 10			T	T	C	T	C	S		67–263	PCR 12	G	T	G				S
	16153–16322	PCR 11			T	T	C	T	C	S		67–263	PCR 13	G	T	G				S
	16153–16320	PCR 12			T	T	C	T	C	S		67–263	PCR 14	G	T	G				S
	16153–16320	PCR 13			T	T	C	T	C	S		69–263	PCR 15	G	T	G				S
	16153–16320	PCR 14			T	T	C	T	C	S		69–263	PCR 16	G	T	G				S
	16153–16322	PCR 15			T	T	C	T	C	S		69–263	PCR 17	G	T	G				S
	16163–16322	PCR 16			T	T	C	T	C	S		69–263	PCR 18	G	T	G				S
	16189–16322	PCR 17			T	T	C	T	C	S		69–263	PCR 19	G	T	G				S

1 rCRS, revised Cambridge Reference Sequence [33]. Skull A, sequence from the putative skull of St. Birgitta 1303–1373. Skull B, sequence from the putative skull of Katarina 1331–1381.

2 L and S denotes PCR products amplified with primers yielding long (440 and 415 bp) or short (221 and 243 bp) fragments (Table 3).

Also, the quality of the sequence differed between the two skulls. Although most sequences were of good quality and easily interpreted, a few sequences revealed a mixture of two nucleotides in a single position. A possible explanation for this is post mortem damage, resulting in base modifications due to hydrolytic or oxidative damage [11]–[14]. This phenomenon of incorrect base incorporation was observed only in a few of the sequences from skull B (8%), while it occurred more frequently in sequences from skull A (17%) (Table 2). To further evaluate the difference observed between the two skulls, the quantity of mtDNA was estimated using a real-time PCR assay based on a mitochondrial target of 143 bp [15]. The two DNA extracts from skull A contained 142 and 31 mtDNA copies per mg bone, while the two extracts from skull B contained 523 and 293 copies/mg. Taken together, there is a difference in PCR success rate, sequence quality and mtDNA content between the two skulls, supporting the theory that there is greater damage of DNA in skull A. There are two plausible explanations for this observation: 1) the two skulls are of different age; and/or 2) the skulls were maintained under different conditions.

**Figure 4:**
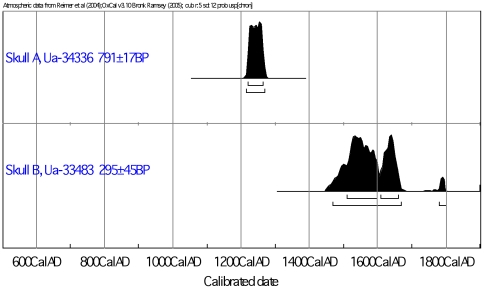
Calibration of radiocarbon ages to calendar dates.

To investigate if the skulls, as indicated by the DNA analysis, could be of notably different ages, a radiocarbon dating was performed. The radiocarbon dating revealed that skull A is from the period 1215–1270 cal AD (2 σ significance), while skull B is from the period 1470–1670 cal AD (2 σ significance) (Table 1 and Figure 4). Thus, a large age difference was confirmed and none of the periods coincide with the time when St. Birgitta and Katarina lived. Although the radiocarbon dating does not support that the skull A relic is from Europe's patron saint–St. Birgitta, we cannot unequivocally eliminate this possibility solely based on the radiocarbon dating. One possible explanation to an older radiocarbon age is the impact from a reservoir effect caused by a food intake of none terrestrial origin e.g. fish from lake or sea environments [16]. An estimate of this reservoir effect can be gained from the stable isotope value, δ^13^C. In the putative skull of St. Birgitta, the measured natural mass fractionation δ^13^C is −18,5‰ (Table 1). An additional stable isotope analysis of nitrogen, δ^15^N, was conducted to distinguish between a pure terrestrial food and fresh water fish intake. The analysis was performed on the collagen bone fraction with the result 12.2 ‰ that is somewhat higher than expected for a pure terrestrial food intake (C∶N ratio  = 3.16±0.08). For comparison δ^13^C and δ^15^N were also measured on collagen from the considerably younger skull B with the results −20.3 ‰ and 12.19 ‰, respectively (C∶N ratio = 2.84±0.04). Although the skull B is from a more recent chronological period than skull A, a similar relatively high δ^15^N value is obtained despite a lower more terrestrial indicative δ^13^C value. It is in this context important to note that a too young age compared to what is expected, which is the case for skull B, can not be explained by a reservoir effect.

## Discussion

Ancient DNA has several characteristics that complicate analysis such as degradation, post-mortem damage, minute amounts, presence of inhibitors and potential contamination by modern DNA. Nevertheless, many successful analyses of ancient materials have been performed. Examples of analysis of ancient materials involve DNA from a Neanderthal fossil, tissue from the mummified Tyroler Ice Man, and bone from the assumed evangelist Luke (AD 150) [17]–[19]. Analyses of more recent remains concern a putative son of King Louis XVI and Marie-Antoinette (1795), the legendary outlaw Jesse James (1882) and the Russian Tsar family, Romanov (1918) [20]–[22]. A major obstacle in ancient DNA analysis is the risk of contamination by modern DNA, which can be high in concentration and often of a better quality than that contained within the remains. Therefore, ancient DNA analysis is constrained with several requirements in order to ensure authenticity. Different criteria have been proposed in order to ensure that the results are genuine [23]–[28]. However, recently it has been proposed that strict adherence to these criteria should not be required as proof of authenticity, rather all available information in each case should be critically considered [29]. Although the criterion to reproduce the results in an independent laboratory is ideal, this can be difficult to arrange due to the fact that the material in many cases are very precious in that they are unique and usually in small amounts [26], [27].

In this study, anthropological and DNA based analysis indicate that the two skulls were derived from female individuals, consistent with the skulls being from St. Birgitta and Katarina. However, the mtDNA results revealed that the two skulls could not be from maternally related individuals. When evaluating DNA results, one always should consider that contamination with modern DNA is a potential explanation for the observed sequence differences. One could suggest that the mixture (two nucleotides in a single position) observed in a few sequences from the skulls is a result of contamination. However, even if the observation was a consequence of exogenous DNA, five of the six positions found to differ (in the hypervariable regions and excluding the HVII c-stretch) between the two skulls did not show any signs of mixture, supporting the conclusion that the skulls of the two individuals in the shrine are not maternally related (16189 showed possible post mortem damage in a few sequences from skull A). All sequences and mixed positions are shown in Table 2.

Typically it is very difficult to obtain amplification products longer than 100 bp from ancient remains and bone material that have been buried for a long time. However, the two skulls in this study appear well preserved, both by visual examination and molecular analysis. The high grade of preservation could be a consequence of the fact that the skulls may not have been buried for more than a short period of time. In addition, a new, more effective extraction method was used which enables recovery of longer and better preserved DNA fragments and a bleach wash was utilised prior to extraction for removal of exogenous DNA [4], [5]. Also, the mtDNA results were different from the analysts in the laboratory who performed the analysis. With all the precautions taken, support for the contamination hypothesis is reduced favouring the hypothesis of no maternal relationship.

Ideally, an additional analysis would be preferred with another sampling from the skulls in an independent laboratory, but that was not possible in this case as the relics are precious due to historical and religious values. For this reason, as well as the indication of the skulls being of different age, an alternative strategy involving radiocarbon dating was chosen to support or refute the DNA results in this study. The radiocarbon dating support that the skulls are of notably different age as skull A is dated to the period 1215–1270 cal AD, and skull B is dated to 1470–1670 cal AD (Table 1 and Figure 4). Therefore, while contamination by modern DNA could have been one explanation for the different mtDNA sequences between the two skulls, the time difference do not support a mother-daughter relationship between the individuals. These data together provide strong evidence that the two skulls cannot be from a mother and daughter who both passed away at the end of the 14^th^ century.

Regarding the dating one has to consider that there could be contamination with other carbon sources that may have affected the result making skull A appear slightly older. Alternatively, the diet of the individual was such that the carbon consumed might make the skull appear older. These potential limitations are not readily resolved. However, it is reasonable to assume that St. Birgitta and her daughter had similar lifestyles and if so could have had similar diets. The reservoir effect can be evaluated by the measured natural mass fractionation of the stable isotope δ^13^C, which in skull A is −18.5 ‰ (Table 1). Although this value is somewhat different from the −20‰ [30] that is expected from a pure terrestrial nutrient intake, natural variations occur in the order of ±2‰ and it is therefore far from definite that a shift in age can be attributed to a reservoir effect. Moreover, the natural carbon mass fractionation (δ^13^C) cannot in a straight and unambiguous manner distinguish between intake of pure terrestrial nutrients and e.g. fractions of fresh water fish. To elucidate this possibility, which might in some cases influence the reservoir effect seriously [31], the corresponding collagen nitrogen mass fractionation (δ^15^N), was also measured. The obtained value of 12.2 ‰ is somewhat higher than the 8–11‰, expected for a pure terrestrial food intake [32]. A large number of factors can contribute to variability in δ^15^N values in human bone collagen e.g. relative quantities of leguminous vs. nonleguminous vegetables, differing manuring practices in arable farming, differential consumption of dairy products, eating different types of meat, eating different types of freshwater fish. It is therefore untenable to directly interpret differences in δ^15^N values as differences in meat or fresh water fish consumption.

**Figure 5:**
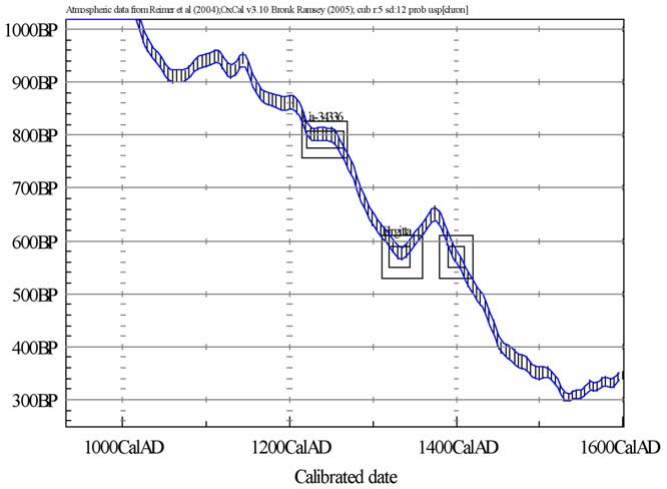
Measured radiocarbon age of skull A (Ua-34336, 791±17 BP) and a simulated radiocarbon age for the time period 1350±20 cal AD corresponding to the life of St. Birgitta (1303–1373 cal AD).

The measured radiocarbon age for skull A is 791±17 BP. If a radiocarbon age is simulated for the time period 1350±20 cal AD representative for the bone from St. Birgitta, a reservoir age of ∼200 years BP have to be assigned (Figure 5). Since a reservoir effect of ∼300 years is considered representative for lakes in Sweden, it implies that if skull A is from St. Birgitta she must have had a diet to large extent dominated by fish. Such diet is questionable in view of the documented traditions in medieval Sweden and due to the fact that St. Birgitta lived in one of the most affluent families in the country during the 14^th^ century. In view of the δ^13^C–δ^15^N results and the inherent interpretation difficulties, it is not possible to completely exclude that skull A can be from the 14^th^ century AD, if an exceptionally high reservoir effect is taken into account. Moreover, if a reservoir effect is present skull A can be from either Katarina or Birgitta as they died within a decade.

In conclusion, the initial maternal relationship hypothesis regarding the two skulls in the relic shrine can be rejected. As always in DNA analysis of ancient remains it is difficult to absolutely exclude that the DNA results may be due to contamination. Therefore, an orthogonal method, radiocarbon dating was used and confirms the exclusion of a mother-daughter relationship demonstrated by mtDNA analysis. Also radiocarbon dating is limited by factors that may have impact on the reliability of the obtained data, which has to be considered. Nevertheless, skull A revealed a date range that is over one hundred years older than that of the time St. Birgitta lived, although this could be attributed to a reservoir effect. Moreover, the radiocarbon analysis provides even stronger evidence that skull B is not from Katarina but rather from a person that lived hundred to three hundred years later.

## Materials and Methods

### Anthropological Investigation

An anthropological investigation was performed to obtain a general overview of the osteologic status of the elements selected for DNA analysis. The sex characteristic features nuchal crest, the supraorbital ridge/glabella and supra-orbital margin were used for sex determination. Skull A was white-grey in colour. Two anomalies were noted. On the left side of the frontal bone a small (approximately 3 mm) delimited osteoma was located. In addition, endocranially a focused thinning of the left parietal bone was observed. The oval concave depression (approximately 25 mm anterioposterior x 24 mm mediolateral) was located about three to four millimetres from the intersection of the sagittal and coronal sutures. Skull B was yellow-brown in colour and except for a small circular elevated bone knob (approximately 8 mm), possibly an osteoma, no pathological changes were noted.

### Prevention of Contamination

Full protective clothing and separated working areas for extraction, amplification, real-time quantification and sequencing setup were used to avoid contamination. Extractions and PCR were performed in separate clean room facilities with HEPA-filtered air, positive pressure and LAF-benches. Furthermore, all working areas and the equipment were regularly UV-irradiated and cleaned with sodium hypochlorite (bleach). Two different analysts performed all steps in the analysis and numerous negative controls were included.

### DNA Extraction

A small piece of each skull was sampled using a saw. DNA was extracted from approximately 1 cm^3^ of bone material. Two extractions were performed from each of the two skulls at separate occasions for purpose of replication. Two pieces from skull A, 0.40 and 0.34 g, and from skull B, 0.48 and 0.56 g bone, were used for extraction. The first extraction involved an incubation of the bone pieces in 6% sodium hypochlorite for 15 minutes for decontamination. It has been demonstrated that relatively well preserved DNA is available in bone within crystal aggregates that are resistant to the NaOCl treatment [5]. This was followed by demineralisation in 2 ml 0.5 M EDTA (pH 8.0). Digestion was achieved by addition of 3 mg proteinase K and incubation for approximately 17 hours at 65°C [4]. Then a salting out procedure was performed for extraction of DNA, according to the Wizard® Genomic DNA Purification Kit protocol, with minor modifications (Promega Corporation, Madison, WI). The second extraction of the two skulls was performed using a similar protocol based on the Wizard® Genomic DNA Purification Kit. The protocol differs from the extraction described above by omitting the sodium hypochlorite treatment. Moreover, the bone pieces were pulverised using liquid nitrogen and the powder was soaked at 37°C in 6 mM EDTA, 5% SDS and 20 mg proteinase K.

### Quantification

A real-time quantification assay, based on a mitochondrial target of 143 bp, was performed as previously described [15]. Undiluted as well as diluted DNA extracts (1∶10) were quantified to assess whether inhibitors were present in the samples. As the highest number of accessible mtDNA copies was seen for the 1∶10 dilution extracts (possibly explained by inhibitors in the extracts), this dilution was used for all PCR reactions.

### DNA Amplification

The hypervariable regions (HVI and HVII) of the mitochondrial genome (mtDNA) were amplified using four different primer pairs, generating short (221 and 243 bp) as well as longer (440 and 415 bp) amplification products (Table 3). In total, 24 amplifications were performed from each of the four extracts (6 shorter HVI, 6 longer HVI, 6 shorter HVII and 6 longer HVII PCR reactions). Two mtDNA coding region fragments were amplified (229 and 223 bp), using primers and cycling conditions shown in Table 3 [10]. The PCR amplification reactions contained 1x PCR Gold Buffer (Applied Biosystems, Foster City, CA), 2.4 mM MgCl_2_, 0.2 µM of each primer, 5 U AmpliTaq Gold DNA Polymerase, 0.2 mM of each dNTP, 0.16 mg/ml BSA, and 10% glycerol in a total volume of 30 µl. To each reaction, 10 µl of diluted (1∶10) DNA extract from the skulls or 1 µl genomic DNA from analysts was added. In general, only 2–4 reactions from the same extract were set-up at the same time together with multiple negative controls.

**Table 3:** Primer sequences and cycling conditions used for amplification.

Name	5′ Primer sequence	DNA region	Size of fragment
**IFb-16128**	GGTACCATAAATACTTGACCACCT	HVI^1^	221 bp
**IR-16348**	GACTGTAATGTGCTATGTACGGTAAA		
**IIFa-45**	ATGCATTTGGTATTTTCGTCTG	HVII^1^	243 bp
**IIR-287**	TTGTTATGATGTCTGTGTGGAAAG		
**15971**	TTAACTCCACCATTAGCACC	HVI^1^	440 bp
**16410**	GAGGATGGTGGTCAAGGGAC		
**L15**	CACCCTATTAACCACTCACG	HVII^1^	415 bp
**R429**	CTGTTAAAAGTGCATACCGCCA		
**2988 F**	CGATGTTGGATCAGGACA	C 2988 F^2^	229 bp
**3216 R-B**	GGGTGGGTGTGGGTATAA		
**16496 F**	GACATCTGGTTCCTACTTCA	NC 16496 F^2^	223 bp
**149 R-B**	ATGAGGCAGGAATCAAA		
**Amelogenin F**	CCCTGGGCTCTGTAAAGAATAGT	Chr X and Y^3^	106 (XX)
**Amelogenin R**	ACTAGAGCTTAAACTGGGAAGCTG		112 (XY)

1 Amplification was performed in a GeneAmp 9700 PCR System (Applied Biosystems) by a 10 min incubation at 95°C, followed by 45 cycles of 301 s. at 95°C, 45 s. at 60°C and 60 s. at 72°C. The program was completed by an extension step at 72°C for 7 min and a final hold at 4°C.

2 Amplification of the coding mtDNA fragments was performed as described above, with an annealing temperature of 53°C instead of 60°C [10].

3 Amplification of the amelogenin gene was performed in a GeneAmp 9700 PCR System (Applied Biosystems). The cycling conditions were 10 minutes at 95°C, 45 cycles of 30 s at 95°C, 45 s at 55°C, 60 s at 72°C and a final extension step for 7 minutes at 72°C.

### Sequence Analysis of mtDNA Hypervariable Fragments

Purification of PCR products were performed using the QIAquick® PCR Purification Kit (Qiagen, Hilden, Germany). Each product was eluted in 60 µl of dH_2_O. Forward and reverse sequencing was performed using the ABI PRISM® BigDye™ Terminator Cycle Sequencing Ready Reaction kit (v.3.3) (Applied Biosystems) and the amplification primers as sequencing primers. Sequence analysis was performed on a 3700 instrument (Applied Biosystems). Data was analysed and compared to rCRS using the Sequencher 4.5 software (Gene Codes Corporation, Ann Arbor, MI) [33].

### Sequence Analysis of mtDNA Coding Fragments

To generate ssDNA, biotinylated PCR product (25 µl) was immobilized to Streptavidine-coated beads (Amersham Biosciences, Uppsala, Sweden), followed by template preparation using the Sample preparation mix (Biotage AB, Uppsala, Sweden). The PSQ™96MA SQA Reagent Kit (Biotage AB) was used for the sequencing reaction. Sequencing was performed in the PSQ™96MA system (Biotage AB), using a directed dispensation order [10]. The pyrograms were analysed and compared to rCRS.

### Sex Determination

A Pyrosequencing based sex determination analysis was performed of the skulls. The assay is based on a 6 bp deletion on the X-chromosome in the amelogenin gene and this target is commonly used in forensic investigations [34]. Amplification was performed in a total volume of 30 µl, containing 1x PCR Gold Buffer (Applied Biosystems), 0.2 mM dNTPs, 1.5 mM MgCl_2_ (Applied Biosystems), 10% Glycerol, 0.16 mg/ml BSA, 0.2 µM of each primer and 5U AmpliTaqGold™ (Applied Biosystems) and 10 µl DNA extract (concentrated using microcon centrifugal Filter Devices YM-30, Millipore). The primers and cycling conditions are listed in Table 3. The analysis was repeated at separate occasions by two different analysts.

### Age Determination

The ^14^C analyses were performed following standard protocols for dating bone samples by collagen extraction and AMS accelerator determination of the ^14^C content with the Uppsala 5MV pelletron system. A standard HCl-gelatine pre-treatment procedure [35] was applied since the bones were of high quality (plastic, light coloured, and released a distinct organic smell upon drilling) and well preserved without any indications of being buried underground. The surface of small pieces of bone from each of the two skulls was mechanically cleaned (scraping, and in some cases sand blasting). The sample was ultrasonically cleaned in boiled distilled water, pH 3, followed by grinding in a mortar. For removal of apatite, 0.8 M HCl was added and the sample was stirred at 10°C for 30 minutes. Distilled water kept at pH 3 was added to the insoluble fraction, which was stirred for 6–8 hours at 90°C. Since the soluble fraction contains most of the organic parts (the collagen) of the original bone, this fraction is dated. The fraction to be ^14^C-dated was combusted by CuO at 800°C for 10 minutes to CO_2_, and then converted to graphite using a Fe-catalyst reaction with H_2_. The natural mass fractionation, δ^13^C, was measured with a VG OPTIMA dual inlet mass spectrometer. For skull A, a mean value from three different sample preparations from two different bone samples was calculated. The nitrogen mass fractionation, δ^15^N, was measured by two different laboratories using continuous flow mass spectrometry and the reported value is an average of these results. The difference between the values reported by the two laboratories was 0.1 ‰. In general, a mean value from measurements of two to three different bone samples was calculated.
